# Single-cell RNA-sequencing resolves self-antigen expression during mTEC development

**DOI:** 10.1038/s41598-017-19100-4

**Published:** 2018-01-12

**Authors:** Ricardo J. Miragaia, Xiuwei Zhang, Tomás Gomes, Valentine Svensson, Tomislav Ilicic, Johan Henriksson, Gozde Kar, Tapio Lönnberg

**Affiliations:** 1 European Bioinformatics Institute (EMBL-EBI), European Molecular Biology Laboratory, Wellcome Trust Genome Campus, Hinxton, Cambridge, CB10 1SD United Kingdom; 2 Wellcome Trust Sanger Institute, Wellcome Trust Genome Campus, Hinxton, Cambridge, CB10 1SA United Kingdom; 3 Centre of Biological Engineering, University of Minho, Campus de Gualtar, 4710-057 Braga, Portugal; 4 Present Address: University of California, Berkeley, USA; 5 Present Address: Turku Centre for Biotechnology, University of Turku and Åbo Akademi University, Turku, Finland

## Abstract

The crucial capability of T cells for discrimination between self and non-self peptides is based on negative selection of developing thymocytes by medullary thymic epithelial cells (mTECs). The mTECs purge autoreactive T cells by expression of cell-type specific genes referred to as tissue-restricted antigens (TRAs). Although the autoimmune regulator (AIRE) protein is known to promote the expression of a subset of TRAs, its mechanism of action is still not fully understood. The expression of TRAs that are not under the control of AIRE also needs further characterization. Furthermore, expression patterns of TRA genes have been suggested to change over the course of mTEC development. Herein we have used single-cell RNA-sequencing to resolve patterns of TRA expression during mTEC development. Our data indicated that mTEC development consists of three distinct stages, correlating with previously described jTEC, mTEChi and mTEClo phenotypes. For each subpopulation, we have identified marker genes useful in future studies. Aire-induced TRAs were switched on during jTEC-mTEC transition and were expressed in genomic clusters, while otherwise the subsets expressed largely overlapping sets of TRAs. Moreover, population-level analysis of TRA expression frequencies suggested that such differences might not be necessary to achieve efficient thymocyte selection.

## Introduction

The adaptive immune system relies on precise discrimination between self and non-self molecules; cells of the thymic epithelia are indispensable for the development of this property. After being selected by cortical thymic epithelial cells (cTECs) for the ability to bind to either class I or class II MHC molecules with appropriate affinity, thymocytes migrate to the thymic medulla and interact with medullary thymic epithelial cells (mTECs). mTECs are a highly specialized cell type, which, by incompletely understood mechanisms of promiscuous gene expression (pGE), express a large number of tissue-restricted antigens (TRAs): proteins otherwise found only in differentiated cell types. The TRA proteins are subsequently degraded to peptides and presented to thymocytes either by mTECs or thymic dendritic cells^1^. This can trigger apoptosis or differentiation to a thymic regulatory T cell (tTreg) in any thymocyte with sufficient binding affinity^2–6^. As a result, the effector T cell repertoire is purged of clones that interact strongly with self peptide-MHC complexes. The best-established factor contributing to pGE is the AIRE protein encoded by the Autoimmune regulator gene (*Aire*). AIRE acts largely independently of DNA sequence, as indicated by its discrepant targets in different cell types^7,8^ and its deletion leads to a polysymptomatic autoimmune disorder Autoimmune polyendocrinopathy-candidiasis-ectodermal dystrophy (APECED)^9^. However, a significant proportion of TRAs have been found to be expressed also in the absence of *Aire*, strongly suggesting the existence of unknown complementary mechanisms^10,11^. Recently, *Fezf2* was identified to induce TRA expression independently of *Aire*^12^ and other such factors are likely to contribute to the generation of TRA repertoires in mTECs.

At the cellular level, the expression of TRAs is highly variable, with individual TRAs in many cases expressed only by a small fraction (1–3%) of the mTECs^10,13–15^. In addition, the expression patterns can be further influenced by the developmental state of the mTECs^16,17^. The development of mTECs has been proposed to originate from bipotent thymic epithelial progenitor cells (bTEP), also giving rise to cTECs^18,19^. The cells destined for the mTEC fate have been shown to progress through a distinct early developmental stage termed junctional TEC (jTEC)^20^. The differentiation and proliferation of mature Aire-expressing mTECs is ultimately promoted by RANKL and CD40L produced by lymphoid cells^21^. Mature postnatal mTECs can be divided into two main subpopulations on the basis of expression levels of CD80 and MHC Class II molecules. However, these subpopulations are intrinsically heterogeneous and their developmental relationships are unclear^22^. Recently, it has been demonstrated by cell fate mapping that upregulation of *Aire* does not mark the last stage of mTEC lifespan. Instead, at least some cells proceed into a post-*Aire* stage, characterised by loss of *Aire* expression, but retention of other markers of maturation^23,24^.

Altogether, the developmental stages in the thymic medulla are still incompletely understood, as are the mechanisms by which TRA expression is gained, and to which extent is it maintained in the post-*Aire* state. The cell-intrinsic and developmental heterogeneity within the epithelial cells, have made these mechanisms difficult to elucidate using population-level approaches. Herein, we have used single-cell RNA-sequencing to systematically dissect the acquisition of TRA expression during mTEC development. This strategy allowed us to interpret TRA expression in the context of an established timeline of mTEC differentiation, in contrast to the previous strategies which have primarily focused on co-expression patterns of TRA genes. In addition, previously published mTEC single-cell datasets^16,25,26^, although biased towards mature *Aire*-high mTECs (MHCII^hi^), were processed and used to corroborate our findings where appropriate.

## Results

### Single-cell mRNA sequencing of mTEC identifies global characteristics of TRA expression

**Figure 1:**
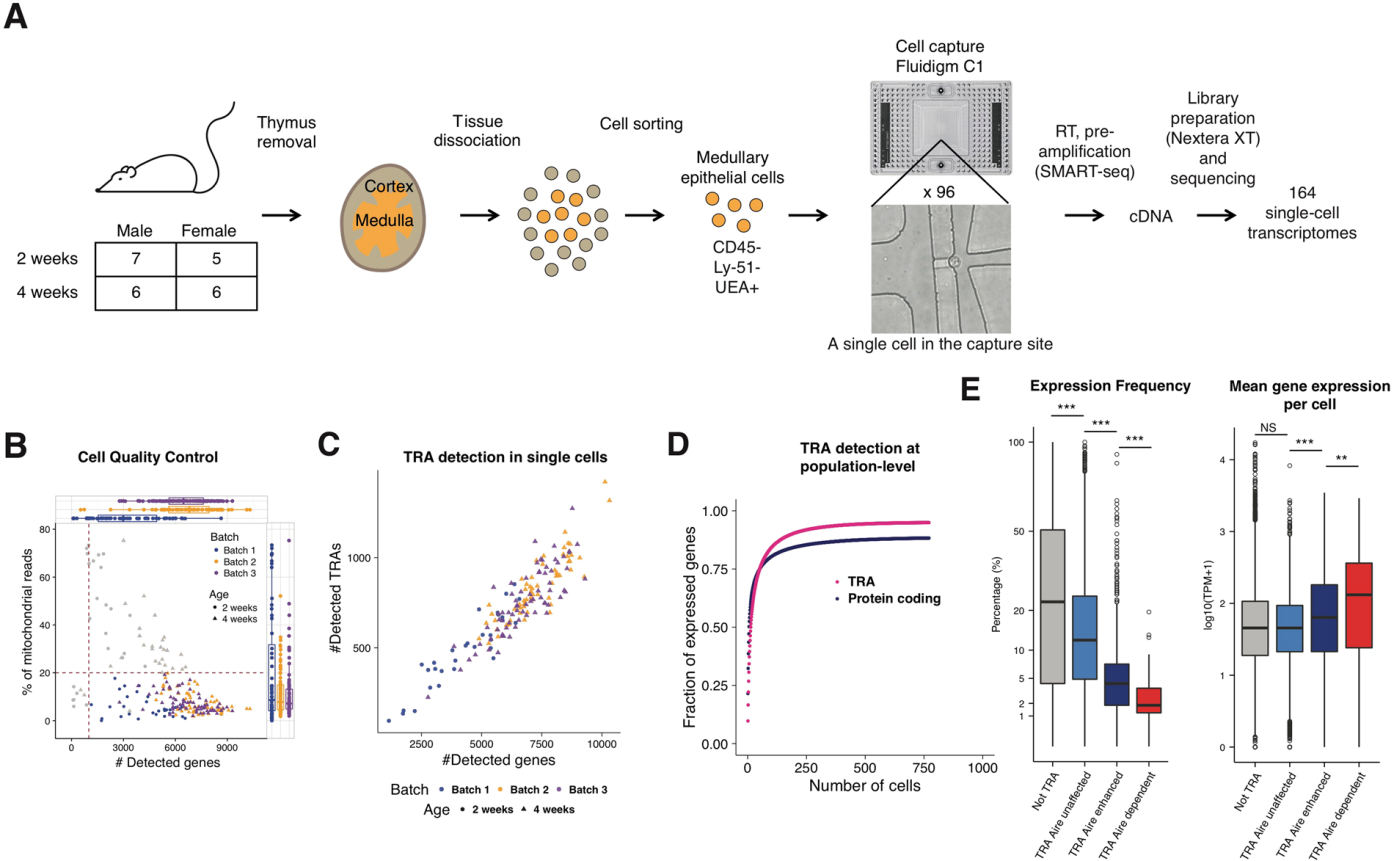
Experimental workflow and expression of tissue-restricted antigens on population level. (**A**) Experimental workflow: mTECs in single-cell suspension were sorted to be run in Fluidigm C1 system. (**B**) Quality control of scRNA-seq for all 3 batches of cells processed with the Fluidigm C1 system based on number of genes detected, percentage of mitochondrial reads and number of mapped reads. Each batch corresponds to a different colour, and the age of the mice used match the shapes. (**C**) Number of expressed genes *vs* number of expressed TRAs in each cell. (**D**) Number of expressed genes as a function of the number of mTECs considered. Each point was calculated based on the average of 100 random orders of the 692 cells of all datasets analysed. (**E**) Comparing genes from different categories in terms of expression frequency and mean expression level across all cells. ***p-value < 0.001, **p-value < 0.01, *p-value < 0.05, NS – not significant, according to Mann-Whitney-Wilcoxon test, p-value adjusted using Bonferroni correction.

To resolve the heterogeneity of the mTEC population, and to dissect the patterns of TRA expression, we sequenced and analyzed the transcriptomes of murine mTECs (PI^−^CD45^−^EpCAM^+^Ly51^−^UEA-1^+^) at the single-cell level (Fig. 1A, Supplementary Figure 1). By using SMARTer chemistry (Clontech) on the C1 autoprep system (Fluidigm), we obtained cDNA libraries from 216 cells, 164 of which met quality control criteria (Fig. 1B, Supplementary Figure 2) and were kept for downstream analysis (Supplementary Tables 1 and 2). Batches of cells coming from different experiments and/or C1 machines were compared by differential expression analysis, and did not show gene expression biases (Supplementary Figure 2B). TRA genes, as defined by Sansom *et al*.^25^ and excluding genes coding for MHCI proteins, totalled 6611 genes expressed in both datasets. On average, TRAs accounted for approximately 10% of all genes expressed in a single cell (Fig. 1C). In line with previous reports^16,25,26^, the repertoires of TRAs expressed in single mTECs did not exhibit significant enrichment for any particular peripheral tissues (Supplementary Figure 3). Integrating publicly available datasets with our data, it is apparent that the majority of protein coding genes, including TRAs, are covered in a couple of hundreds of mTECs (Fig. 1D), also supporting previous observations^4,16^. Further interactions of thymocytes with additional mTECs would therefore result in minimal increase in the variety of TRAs they are exposed to.

To achieve greater resolution, we then divided the TRAs into subsets of genes, of which expression is either completely dependent on *Aire* (*Aire*-dependent), enhanced by it (*Aire*-enhanced), or unchanged in its absence (*Aire*-unaffected) according to previous data in *Aire*-deficient mice^25^. Analyzing the expression frequency and expression level separately for the genes of each of these subsets evidences *Aire*-dependent genes as a notably distinct group, with significantly lower expression frequency and higher mean expression level than genes in the other subsets (Fig. 1E), as previously reported^16,25,26^. Despite the differences between subsets of *Aire* genes, it is worth noticing that all of them were expressed on average at equal or higher levels than all other genes in mTECs. This indicates that *Aire* genes of all subsets, especially *Aire*-dependent, are actively expressed and not merely products of passive and/or residual level expression. We then investigated the behaviour of TRAs controlled by the recently discovered *Fezf2* regulator^12^ (Supplementary Figure 4). In contrast to *Aire*-induced TRAs, *Fezf2*-induced TRAs were expressed as frequently as normal genes (“non-TRA”), although their expression level was higher, similarly to *Aire* TRAs (Supplementary Figure 4). Such differences are likely to stem from the different mechanisms of gene activation by these two transcription factors.

### scRNA-seq resolves three major subpopulations along mTEC differentiation

**Figure 2:**
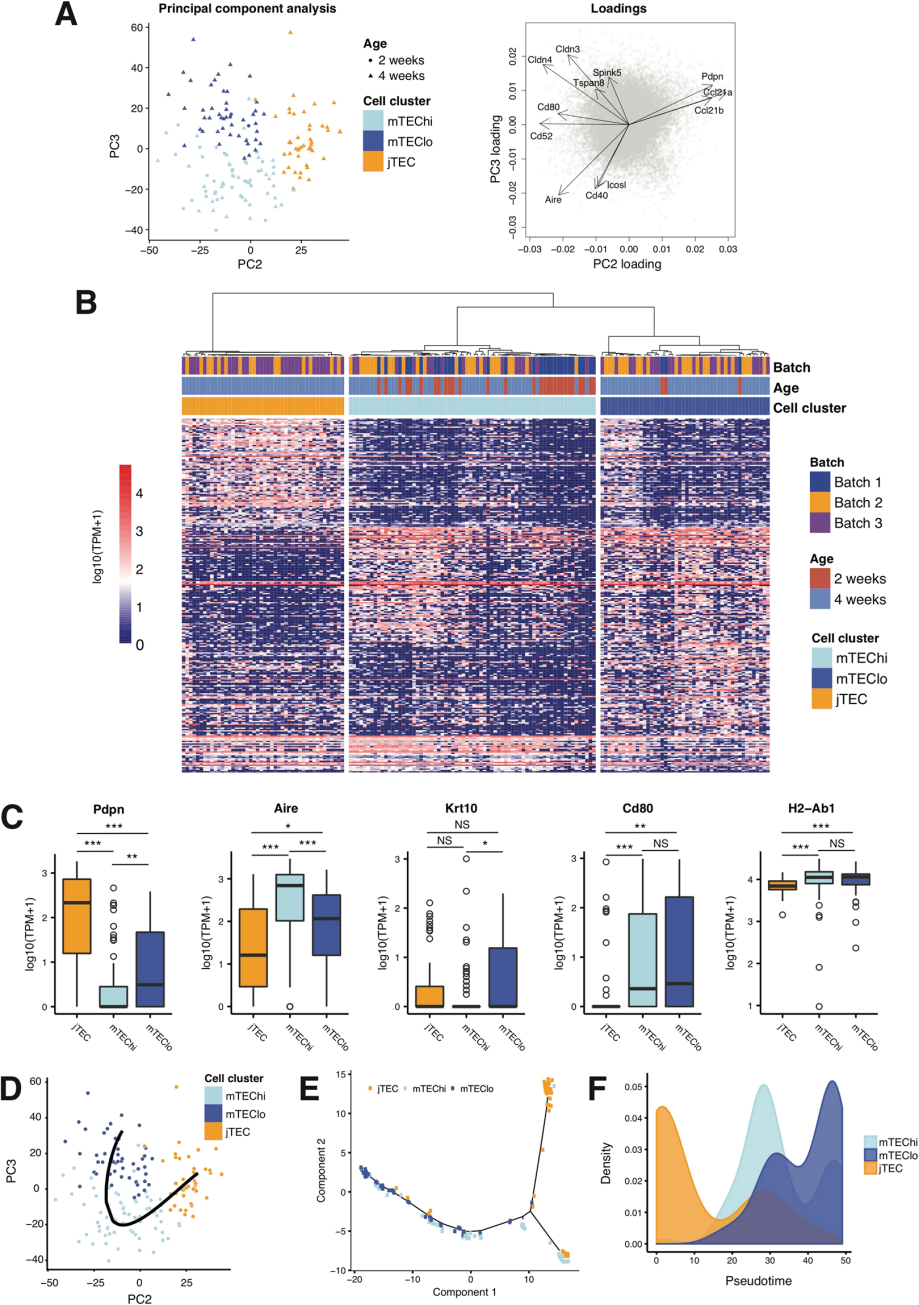
Analyses of mTEC subpopulation structure. (**A**) Principal Component Analysis (PCA) of mTECs using all genes. Batches match colour and age of mice matches shapes (left). The PC2 and PC3 loadings of key genes of interest are highlighted (right). (**B**) Hierarchical clustering of 164 transcriptomes of single-cells, based on the top positively and inversely correlated genes with PC2 and PC3. Three clusters of cells were identified (jTEC, mTEChi and mTEClo). Cell cluster, mice age and batch are depicted. (**C**) Expression of selected marker genes in the jTEC, mTEChi, and mTEClo populations. ***p-value < 0.001, **p-value < 0.01, *p-value < 0.05, NS – not significant, according to Mann-Whitney-Wilcoxon test, p-value adjusted using Bonferroni correction. (**D**) Ordering of single cells in pseudotime by degree of maturation using the pseudogp algorithm. Data points represent single cells, colours denoting cell clusters (**B**). (**E**) Ordering of single cells in pseudotime using the Monocle2 algorithm. Data points represent single cells, colours denoting cell clusters (**B**). (**F**) Distribution of single cells of each cluster (**B**) along Monocle2 pseudotime (**E**).

We performed principal component analysis (PCA) to explore the subpopulation structure within mTECs (Fig. 2A). We noticed that a great source of variability came from cell size (number of detected genes), which correlated strongly with the most important PC1 (Spearman rho 0.92, Supplementary Figure 5A). We thus focused on the next two PCs, markedly less affected by this variable (Supplementary Figure 5A–C). Importantly, cells that were isolated from different mice and processed on different C1 integrated fluidic circuits were dispersed among each other, suggesting that batch effects did not contribute significantly to overall heterogeneity (Supplementary Figure 5B). In addition to cell size and batch, single-cell RNA-seq data can be profoundly affected with variation associated with cell cycle^27^. To assess how much our data was biased by cell cycle, we used the Cyclone package to assign single cells into cell cycle phases^28^ (Supplementary Figure 5D). All but six mTECs were in G1/G0 phase, suggesting relatively modest cell cycle effects over gene expression in this population. Nonetheless, we investigated this further by running scLVM package to evaluate and regress out any cell cycle related biases. We observed that when performing PCA on the scLVM-corrected data, PC1, PC2 and PC3 all correlated with the cell size to some extent (Supplementary Figure 5A, bottom), in contrast with the uncorrected data where cell size appears limited to PC1 (Supplementary Figure 5A, top). Simultaneously, scLVM-corrected PC1 was highly correlated with the original PC2 and scLVM-corrected PC2 with the original PC3 (Supplementary Figure 5E). Therefore, on this particular case in which cell cycle effect is minimal, technical effects could be more easily deconvoluted from biological variability simply by focusing on higher components on the uncorrected dataset. For the publicly available datasets in which cell cycle seemed to have a stronger effect, scLVM correction was adopted.

We observed that PC2 scores of single cells correlated with the expression of several established markers of thymic development, such as *Cldn3&4*, *Pdpn* and *Cd80*^20,21,29^ (Fig. 2A, Supplementary Figure 6). On the other hand, among the top PC3 loadings were *Aire*, *Cd40*, *Icosl* and other genes associated with the mature mTEC phenotype. This further suggested that the variability associated with mTEC development was primarily distributed along these two components. Using the expression data from top PC2 and PC3 genes (loadings above 0.02 and below −0.02), we used a consensus clustering approach that assigned single cells to three distinct sub-populations (Fig. 2B and Methods). This approach did not seem to be influenced by cell size (Supplementary Figure 7A). An initial inspection of established marker genes painted a general picture of the identity of these subpopulations. The cells in the first of these clusters were negative for, or expressed only low levels, of both *Aire* and *Cd80*, and were characterized by the expression of *Pdpn*, a marker of a recently identified population of junctional TEC precursors (jTEC), which gives rise to fully differentiated mTEC^20^. The different levels of *Aire* expression between the second and third clusters led us to classify them as *Aire*-high mTEC (mTEChi) and *Aire*-low mTEC (mTEClo). The cells of the mTEClo population, although resembling a post-*Aire* state for their lower expression of *Aire* and higher expression of Keratin 10 (*Krt10*)^23,24^, expressed similar levels of *Cd80* and HLA (class II) genes as the mTEChi cells (Fig. 2C).

We did not observe statistically significant differences for other proposed markers of mTEC stages (i.e., *Gp2*, *Gad1*, *Ceacam1*, *Tspan8*)^16,17^, although *Gad1* and *Tspan8* tend to characterize mTEChi and mTEClo, respectively (Supplementary Figure 7B), which is in line with previous reports^17^. Notably, no cells from the 2-week old mice fell into the jTEC population, potentially resulting from age-associated changes in properties or frequencies of thymic subsets^30–32^. It is also possible that the limited number of cells from the week 2 mice did not include any such cells due to stochastic effects.

To confirm the robustness of these findings, we sequenced additional cells using the Smartseq. 2 protocol^33^. In addition, we used two recently published single-cell datasets^16,25^, which we processed using the same pipeline and QC parameters used for our original data (Supplementary Figure 2C). Importantly, cell clustering and expression of marker genes remained largely consistent across these datasets (Supplementary Figures 8 and 9), despite Brennecke *et al*. and Sansom *et al*. enrichment for MHCIIhi cells. This sorting strategy difference is reflected in the proportion of jTEC, mTEChi and mTEClo obtained (C1: 28/43/29%, Smartseq. 2 in-house: 12/36/53%, Brennecke: 26/51/23%, Sansom: 27/62/11%), but it is still permissive enough to include cells from all three subpopulations.

To further investigate this differentiation process, we infer pseudotime, which represents a measure of cells progression along the differentiation trajectory. First, based on the data in reduced dimension as shown in Fig. 2A, we applied pseudogp^34^ and obtained the trajectory shown in Fig. 2D. The inferred curve showed a pattern of differentiation from jTEC to mTEChi to mTEClo. We then used a diffusion map based non-linear dimension reduction implemented in Destiny^35^ for pseudogp, and also got a clear trajectory showing the same jTEC-mTEChi-mTEClo pattern (Supplementary Figure 10A). Finally, we used Monocle2^36^ which does not take our specified dimension reduction result to infer the pseudotime (Fig. 2E). Monocle2 suggested a trajectory consistent with the results from pseudogp, where jTEC positioned towards the beginning of the pseudotime, followed by mTEChi and ending with mTEClo cells. mTEChi and mTEClo overlapped to some extent, suggesting a close relationship between them (Fig. 2E and F). In contrast, the clear gap between jTEC and mTEC indicated more profound differences between these subpopulations.

**Figure 3:**
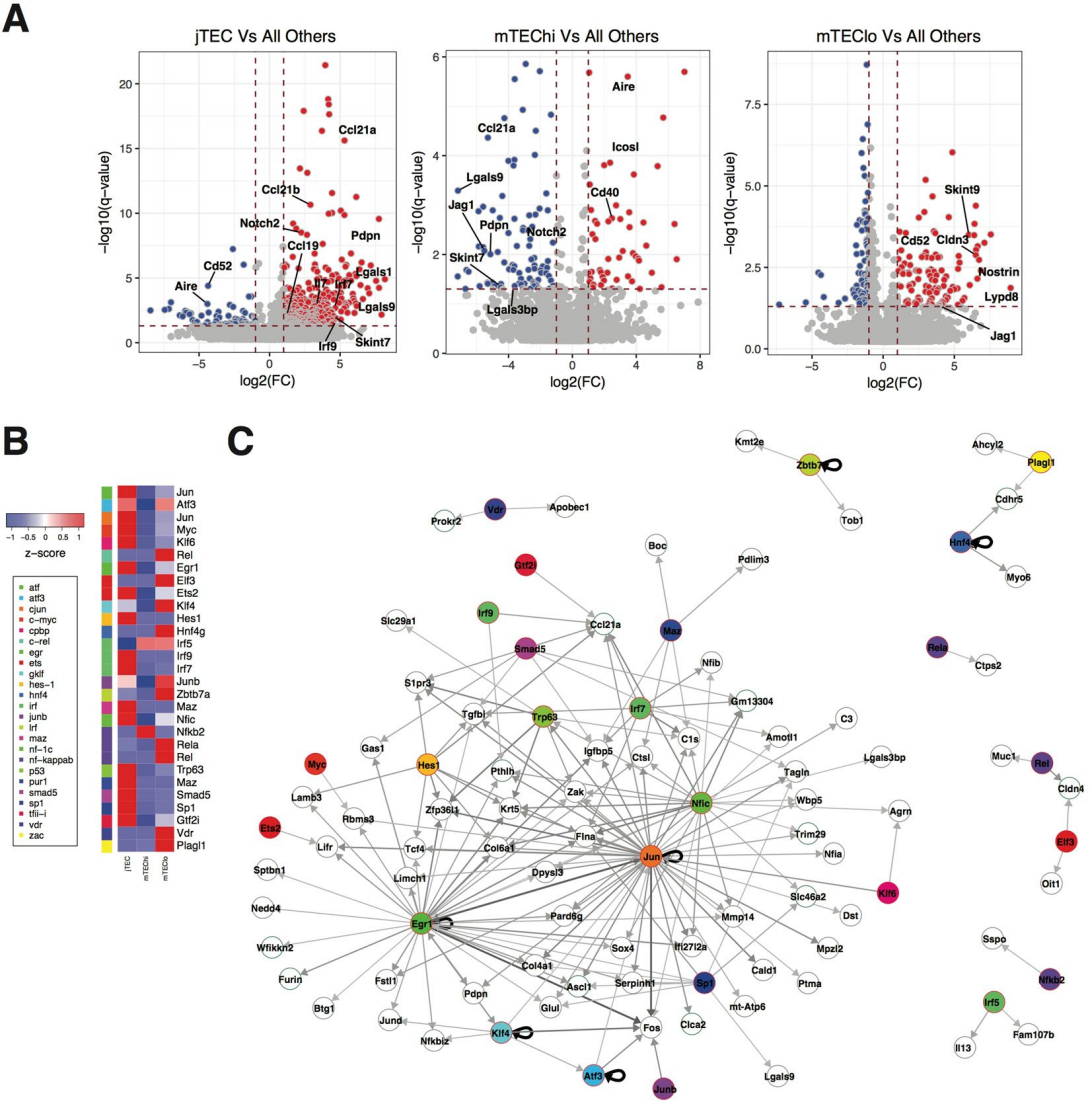
Differential expression of genes across mTEC subpopulations. (**A**) Genes differentially expressed (DE) between each cell population and the remaining populations. The significance of this DE was calculated using a linear model. q-value < 0.01 and |FC| > 1. (**B**) Scaled median expression levels of transcription factors with enriched binding motifs (**C**) across jTEC, mTEChi and mTEClo subpopulations. The TFs were grouped according to TF families, as denoted by the color bar. (**C**) Network visualisation of TFs and their putative target genes. Target genes were identified by binding motif analysis using gProfileR (Methods). The results were further filtered based on co-expression, based on Spearman correlation and Jaccard index (Methods). Colours of the nodes denote TF families, as shown in (**B**). Thicker and darker edges represent higher Spearman correlation.

We then performed differential expression analysis to systematically identify genes that are specific to each subpopulation (Fig. 3A and Supplementary Figure 10B). The jTEC state was associated with upregulation of 383 genes and downregulation of 63 genes (*q*-value < 0.05, |log2(FC)| > 1). In the mTEChi cells, 50 genes were upregulated (including *Aire*) and 90 were downregulated. The mTEClo population was characterised by 109 upregulated and 81 downregulated genes. It is worth mentioning that of the genes differentially expressed between the mTEC subpopulations, only 45 encoded for TRAs. Notably, almost all of these (43) were *Aire*-unaffected TRAs. The notable absence of *Aire*-regulated TRAs among these genes is probably explained by their relatively lower expression frequency. Furthermore, some of the jTEC-specific TRAs (such as *Adm*, *Cdh3*, *Krt14* and *Krt17*) are associated with epithelial development, and are thus likely to be required for a specific functional role despite being been considered TRAs.

Several of the markers now identified seem to be related to particular states of maturation, interactions with the surroundings and/or specific functions of each subpopulation. For example, *Jag1* upregulation by mTEChi cells, together with *Notch2*, *Hes1* and *Hes6* upregulation in jTECs suggest that Notch signalling might be involved in the jTEC to mTEC transition. Based on the expression of genes such as *Jag1*, *Cd40* and *Icosl* in mTEChi^37–39^, Skint-family genes (*Skint7* and *Skint9*)^40^, galectins and related genes (*Lgals1*, *Lgals9*, *Lgals3bp*) in jTEC and mTEClo^41,42^, the way each subpopulation instructs thymocytes is likely to be slightly different, *e*.*g*. role of ICOSL in the expansion of regulatory T cells (Treg) in humans^39^. From a practical point of view, membrane proteins in these sets of marker genes (Supplementary Table 3) can potentially be used in the future to sort out each subpopulation for further studies (e.g. *Lypd8* to distinguish mTEChi and mTEClo).

### Binding motif and co-expression analysis highlights potential drivers of mTEC maturation

Our transcriptomics data revealed three distinct stages during mTECs lifetime. Considering the importance of mTEC development for central tolerance, we wanted to identify transcription factors (TFs) promoting this process, as well as their respective target genes. To address this question, we focused on the top PC2 and PC3 genes, which were also used for cell clustering (Supplementary Table 4). We selected the TFs within this set of genes, most of which were preferentially expressed in one of the three mTEC subpopulations (Fig. 3B). We then scanned the genomic regions upstream (1 kb) the top PC2 and PC3 genes in search for binding-sites for any of these TFs. Genes possessing a binding-site for a given TF were then considered as being potentially regulated by it. The gene-to-gene correlation between TF and respective target was then calculated for all TF-target pairs. The most significant TF-target relationships were filtered using stringent correlation and Jaccard index threshold values (Methods). These relationships were thus considered to potentially indicate direct regulation of the target by the TF, and were visualised in as a co-expression network (Fig. 3C).

In this network, the most prominent TF hubs were *Egr1* and *Jun*. Although both are characteristic of jTEC, the program they set in motion is likely to also span mTEChi and mTEClo (*e*.*g*. *Klf4*). *Ccl21*, a key marker of the jTEC subpopulation, was identified as a putative target of seven different TFs. Notably, *Nfκb* and *Irf* transcription factor families were well represented across all three subpopulations: jTEC expressing *Irf7* and *Irf9*, mTEChi expressing *Irf5* and *Nfκb2*, and mTEClo expressing *Irf5*, *Rel* and *Rela*. Both classical and non-classical NF-κB signaling (through TRAF6 and NIK, respectively) have been proven necessary for the development of *Aire*-positive mTECs^43^. More recently, the *Irf* family has also been implicated in the development of mTECs^2^ and shown to contribute to TRA expression along with AIRE^8^. *Hes1* expression by jTEC and *Zbtb7a*^44^ by mTEClo suggest the involvement of the Notch pathway in this progression. Finally, *Vdr*, *Plagl1*, *Zbtb7a*, *Hnf4g*, most of which have previously been detected in mTEC^17,45,46^, assume particular relevance as presumptive drivers of the late stages of mTEC differentiation.

### TRA expression during mTEC development

We next sought to further investigating TRA expression patterns across the mTEC subpopulations. Several models have been put forward in efforts to explain how TRA expression is regulated during mTECs lifetime to guarantee a comprehensive negative selection of self-reactive thymocytes. Do mTECs progressively express a higher number of TRAs as they differentiate (“terminal differentiation model”)? Do they begin with the capacity to express significant numbers of TRAs, and then progressively (and independently of other mTECs) limit the range of TRAs expressed (“progressive restriction”)^47^? Are certain sets of TRAs co-expressed, or in a predefined sequence^16^?

**Figure 4:**
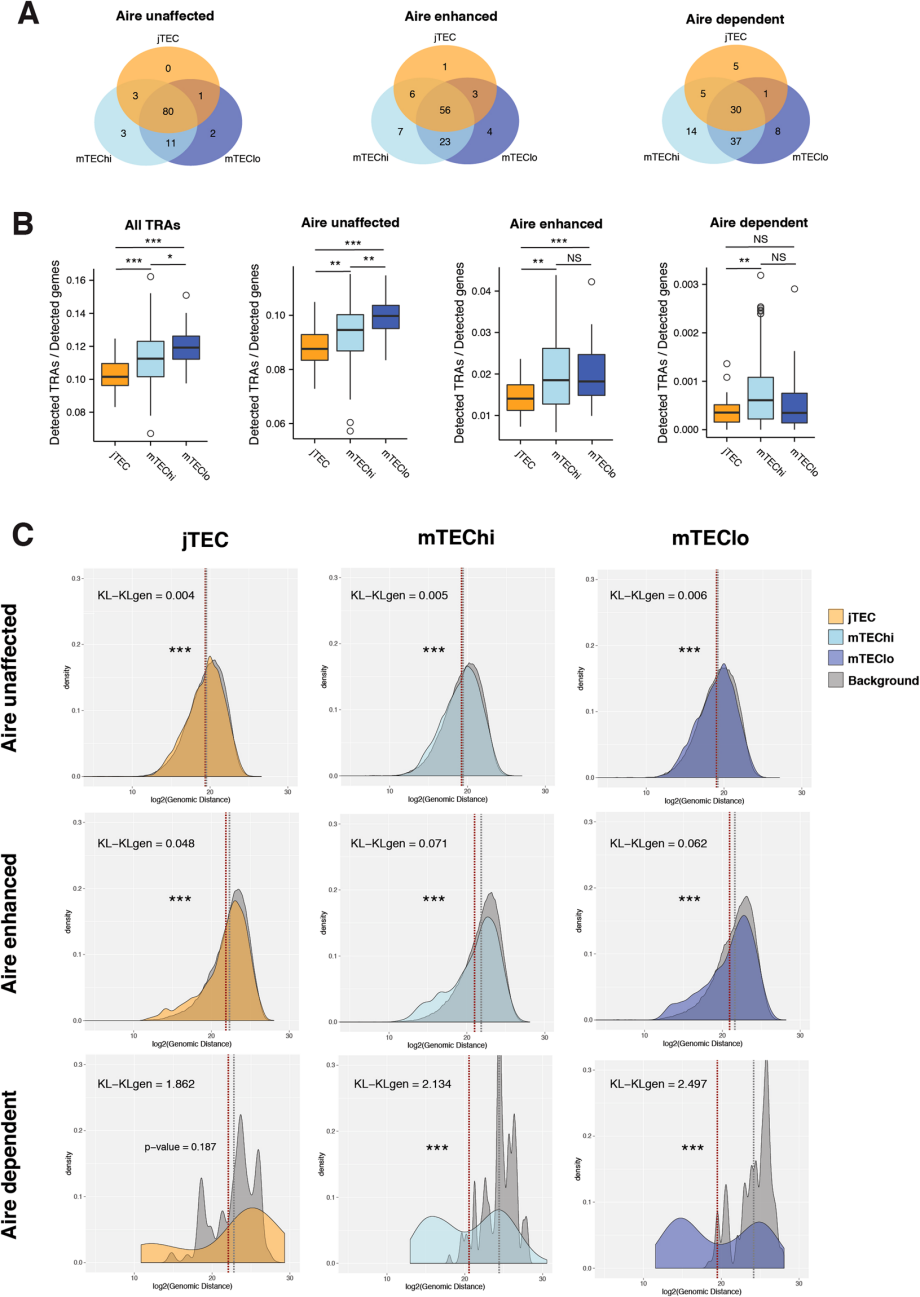
Expression of TRAs across mTEC subpopulations. (**A**) The detection of TRA genes across jTEC, mTEChi, and mTEClo populations. The numbers indicate the fraction of TRAs detected in the respective subset of cells. (**B**) Number of TRA genes expressed in the jTEC, mTEChi, and mTEClo populations. To account for differences in library sizes, the number of detected TRA genes were normalised to the number of detected genes per cell. ***p-value < 0.001, **p-value < 0.01, *p-value < 0.05, NS – not significant, according to Mann-Whitney-Wilcoxon test, p-value adjusted using Bonferroni correction. (**C**) Genomic distribution of expressed TRAs. Histograms represent distribution of distance to the nearest neighbour gene for TRA genes. The background histograms represent the distribution of distances to the nearest neighbour of randomly sampled expressed genes from a control distribution (Methods) in the corresponding cell.

With these questions in mind, we sought to investigate the extent to which the mTEC subpopulations differ in their TRA expression patterns (Fig. 4A). Very few genes (in any of the TRA-subsets) were uniquely expressed by jTECs, in line with the notion that they are the most immature population. Surprisingly, jTECs covered as many as 84% of *Aire*-unaffected TRAs and the majority (66%) of *Aire*-enhanced TRAs. Nonetheless, the percentage of *Aire*-unaffected TRAs and *Aire*-enhanced TRAs shared exclusively between mTEChi and mTEClo (11% and 23%, respectively) was larger than between either population and jTECs. *Aire*-dependent TRAs were expressed by jTEC to a lesser extent (41%) than the other TRAs, and once again, mTEChi and mTEClo shared 37% exclusively between them and expressed a higher percentage of unique genes (14% for mTEChi and 8% for mTEClo) (Fig. 4A). Together, these observations indicate that the TRA repertoires are largely overlapping between the three maturation stages, except for some compartmentalization of *Aire*-dependent TRAs.

Next, we wanted to assess the performance of individual cells within these groups. To measure their competency in driving the negative selection process, we measured the level of expression and the number of TRAs expressed by individual cells in jTEC, mTEChi and mTEClo. As expected, mTEChi expressed significantly elevated levels of *Aire*-enhanced and *Aire*-dependent TRAs (Supplementary Figure 10C). Then, we assessed the number of TRAs expressed on a cell-by-cell basis, normalized by the number of detected genes, thus accounting for differences in sequencing efficiency of single cells (Fig. 4B). While the jTEC stood out as the least competent subpopulation, they covered a surprisingly high fraction of TRAs, which is especially interesting for Aire-dependent TRAs given the relatively low expression of *Aire* in jTEC. However, jTEC performed significantly worse in terms of expression efficiency at single-cell level, which explains the low levels of Aire-dependent genes detected in previous studies^20^.

The mTEClo were at the other end of the spectrum, expressing the highest number of TRAs per cell. Specifically, mTEClo expressed the highest number of *Aire*-unaffected TRAs, and a similar number of *Aire*-enhanced and even *Aire*-dependent TRAs compared to mTEChi (Fig. 4B) an equivalent number of *Aire*-enhanced TRAs compared to mTEChi. mTEChi seem to perform slightly better with respect to *Aire*-dependent TRAs, although the advantage over mTEClo is not significant. Genes controlled by *Fezf2*, a master regulator of TRAs besides *Aire*^12^, were shown to increase consistently in number and level of TRA expression along differentiation (Supplementary Figure 10D).

In summary, we observed that all three subpopulations of mTECs collectively expressed most of the TRA genes. However, they did differ in their expression efficiency in terms of level and number of TRAs expressed, with mTEChi holding an advantage for *Aire*-regulated TRAs, as expected. Nevertheless, mTEClo, a state that has been classically regarded as a passive step towards mTEC death, showed remarkable competence at expressing *Aire*-enhanced and *Aire*-unaffected TRAs. These observations are largely confirmed in other datasets (Supplementary Figure 11). They are also in line with previous reports^24^ and indicate that mTEClo are active players in negative selection, with TRA expression increasing during the entire mTEC lifetime and remaining high after *Aire* expression declines.

### Expression of *Aire*-dependent TRAs is associated with strong genomic enrichment at single-cell level and is induced during jTEC-mTEC transition

About a decade ago, *Aire*-regulated genes were shown to be in close linear chromosomal proximity to each other, forming genomic microclusters^10,48^. Since then, it became accepted that AIRE’s ability to recruit transcription factors to regions of closed chromatin would induce remodelling of such segments, thus facilitating the co-expression of neighbouring genes^49^. The discovery that AIRE binds super-enhancers^50^ supports this idea, providing a model that explains both intra- and interchromosomal coexpression patterns of *Aire*-regulated genes^26^. We became interested in further investigating this gene clustering effect at the single cell level in order to determine how it changes during mTEC lifetime, how differently it affects *Aire*-enhanced and *Aire*-dependent TRAs, and whether these principles can be generalized to all TRAs.

Thus, we investigated whether mTECs, considering each cell individually, indeed tend to express genes that sit closer together along the genome. We focused our attention on the set of TRA genes and its sub-groups based on *Aire*-regulation and we considered jTEC, mTEChi and mTEClo separately. For each expressed TRA, we calculated the base-pair distance to the nearest expressed TRA on a cell-by-cell basis. The resulting distribution of distances was then compared to a control distribution built to account for the genomic location of TRA genes, as well as the generic clustering effect reported for any set of expressed genes^51^ (see Material and Methods). We compared not only the mean value for the actual and the background distances (*p*-value from Mann-Whitney-Wilcoxon test), but also the magnitude of the divergence between these distributions (Kullback-Leibler divergence (KL)) (Fig. 4C). Although statistically significant, it is clear that genomic clustering of *Aire*-unaffected TRAs is extremely weak for all three subpopulations (KLs ≤ 0.006). For *Aire*-enhanced TRAs, the clustering effect does increase (KLs ≤ 0.071) remaining quite modest nonetheless, particularly for jTECs (KL = 0.048). *Aire*-dependent TRAs, on the other hand, exhibit a distribution of distances indicating strong genomic clustering profile in both mTEChi and mTEClo (KL = 2.134 and 2.297, respectively). In turn, for jTECs there is no statistically significant difference between the *Aire*-dependent TRA distance mean and the background, and the divergence between these two distributions (KL = 1.862) is lower than mTEChi and mTEClo counterparts.

For *Aire*-enhanced TRAs, the majority of observed distances overlap the control, suggesting that for most genes the induction of expression mediated by *Aire* does not rely on the activation of stretches of chromatin. Nonetheless, there is a minor proportion of shorter distances hinting that such a mechanism might be important for a reduced number of *Aire*-enhanced TRA genes. Finally, for *Aire*-dependent TRA genes, the distribution of real distances is markedly distinct from the control background, exhibiting a clear peak of short distances.

Overall, these results are evidence that the genomic clustering tendency affects only a minority of TRA genes. They also highlight that such mechanism preferentially affects *Aire*-dependent genes in comparison to *Aire*-enhanced genes. Finally, this clustering effect seems to be established only during the progression from jTEC to mTEC, as both the number of TRAs per cell and genomic clustering of *Aire*-dependent TRAs were very limited in the jTEC population.

## Discussion

The relevance of mTECs for immune tolerance has granted this cell type close attention in the past decades, with particular emphasis on the expression regulation of TRAs. Until recently, these attempts were severely impaired by the lack of single-cell resolution and by the heterogeneous nature of this set of genes. The recent development of single-cell transcriptomics has finally enabled systematic deconvolution of such complex gene expression patterns. In a short time, this has provided new insights into the biology of mTECs and allowed revaluation of existing models and notions (present work and^16,25,26^). In summary, we approached our data by first identifying differentiation stages within murine mTECs (EpCAM^+^Ly51^−^UEA-1^+^), and then characterizing these stages in terms of TRA expression at the population and single-cell level. With this methodology, we identified three distinct stages of maturation: early mTEC in the cortex-medulla junction (jTEC), *Aire*-expressing mTECs (mTEChi), and mTECs entering the post-*Aire* stage (mTEClo)^20,24^. For each of the three stages we provide markers that can potentially reduce the need for transgenic mice or intra-cellular staining of AIRE in future studies, namely for mTEClo and mTEChi sorting. While our pseudotime analysis indicated that in general, mTEChi stage preceded mTEClo, there was some overlap between the two populations. This suggested that the transition between these states might not be a tightly programmed event and individual cells might undergo it at asynchronous rates. Notably, hierarchical clustering did not clearly segregate cells expressing high or low levels of the widely-used maturation markers *Cd80* and MHC Class II. This might relate to the fact that CD80^low^ mTECs appear to represent a mixed population, containing both immature precursor cells and terminally differentiated post-*Aire* mTECs^22,52^. Besides the expected *Nfkb* and *Irf* families of genes, the regulatory network inferred for mTEC development (Fig. 3C) puts forward candidate TFs as drivers of this process: *Egr1* mainly on jTEC, and *Vdr*, *Plagl1*, *Zbtb7a*, *Hnf4g* on mTEClo.

While the mechanisms and dynamics of mTEC development remain under active investigation, recent reports have strongly suggested that different progenitor populations might play the leading role in perinatal and adult mice^22^. In embryos and during early life, the main contribution seems to be from β5t^+^ precursors with cTEC-like properties^53–55^, or from *bona fide* cTECs^31^, whereas in older mice most mTECs are likely to arise from an intermediate population of lineage-committed cells, sometimes referred to as “transit-amplifying cells”^56^. This shift coincides with the emergence of a population of TECs expressing low levels of CD80 and MHCII^30,31^. In this work, we studied cells from 2- and 4-week old mice, with none of the week 2 cells fell into the jTEC cluster. It would be tempting to speculate that this difference reflects age-associated changes in thymic compartmentalization, with jTECs representing a pool of transit-amplifying cells that becomes more important over time as embryonic-derived progenitors are gradually depleted^32^. At the same time, it remains possible that our data is influenced by random effects associated with limited sample sizes, especially taking into account the lower frequency of CD80/MHCII-low cells in young mice^31^. Notably, while *Pdpn* transcripts were detected in some week 2 cells, these cells did not express *Ccl21*, another marker of jTEC phenotype^20^. Thus, it seems also possible that the properties of the jTECs evolve with age. The exact relationship of the jTECs with the other immature TEC populations remains to be elucidated, along with their exact role in development and homeostasis of the thymus.

Historically, mTEChi, as key expressers of most TRAs and being particularly competent in antigen presentation, have been considered the main player in negative selection in the thymus, while pre- and post-*Aire* stages would be of limited relevance. However, our observations indicate that mTEClo, and to a more limited extent, jTEC cells, might also contribute to this process. The competence of mTEClo in terms of number of TRAs expressed per cell was in fact equal or greater than that of mTEChi (Fig. 4B), suggesting that mTECs progressively express more TRAs as they mature, even after *Aire* expression declines. Moreover, the expression levels of *Fezf2*-affected TRAs were progressively higher from jTEC, to mTEChi and then to mTEClo (Supplementary Figure 10D). In parallel, differentially expressed genes like *Jag1*, *Cd40*, *Icosl*, Skint-family genes and galectins suggested distinct functions/interactions of mTEC subsets during thymocyte development. Overall, these observations were in line with Metzger *et al*.^24^ inspection of MHCII and individual TRA expression in post-*Aire* mTECs, consolidating mTEClo as a key stage in the maintenance of central tolerance. In terms of TRA repertoire, even jTEC could cover most of the *Aire*-unaffected and the *Aire*-enhanced genes, and most TRAs in general (Fig. 4A). However, the cell-to-cell ability to express them was significantly impaired (Fig. 4B), which likely explains the low levels of TRAs detected in previous reports, namely for *Aire*-dependent TRAs^20^. Taken together, our results indicated that while AIRE is important for turning on TRA expression during jTEC-mTEC transition, the TRA expression was maintained in mTEClo even in the absence of AIRE. In summary, AIRE seems to be critical for inducing TRA expression, but not for maintaining it.

Theoretically, a couple of hundred mTECs are enough to collectively express the entire repertoire of TRA genes (Fig. 1D and ref.^16^), and further contacts of thymocytes with additional mTECs would therefore be unnecessary for increasing their exposure to new self-antigens. This number of mTECs fits perfectly with the observation that thymocytes visit only a small number of confinement areas (each containing 100–200 mTECs)^57^, and appears to be a highly energy/time-effective strategy for covering as much of the TRA repertoire possible. In line with this scenario, we observed little divergence between the TRA repertoires of each maturation stage (Fig. 4A), meaning that the TRAs encountered by thymocytes anywhere across the medulla should not depend largely on the maturation stage of the surrounding mTECs. This is particularly relevant as mTECs tend to re-locate to different regions of the medulla during their maturation (jTEC in the cortex-medulla junction, mTEChi towards the periphery of the medulla and mTEClo towards the centre of the medulla)^24^. Nonetheless, we cannot exclude the possibility that we have overlooked subtle differences along the differentiation process (*e*.*g*. *Tspan8* and *Gad1* trends (Supplementary Figures 7B and 9B), or that subtle TRA biases are present within each subpopulation of mTECs. It remains possible that such patterns can be elucidated by future studies employing technologies with higher cell throughput and higher transcript detection sensitivity. In addition to cell number and transcript detection sensitivity, our scRNA-seq approach had several limitations that could potentially be addressed by complementary techniques. Firstly, mRNA quantities do not necessarily correlate linearly with protein quantities, and methods for parallel measurements of these at single cell level are emerging^58–61^. Secondly, as scRNA-seq represents a temporal snapshot, each cell is sampled only once and it is not possible to infer direct developmental relationships. Finally, our approach did not record any spatial information. Methods for interrogating single cells in a spatial context have been recently developed^62,63^, and might provide valuable information on the influence of thymic location (inner vs. outer medulla, cortico-medullary junctions) on cellular phenotypes.

## Methods

### Ethics statement

C57BL/6 mice were maintained under specific pathogen-free conditions at the Wellcome Trust Genome Campus Research Support Facility (Cambridge, UK). These animal facilities are approved by and registered with the UK Home Office. All procedures were in accordance with the Animals (Scientific Procedures) Act 1986. The protocols were approved by the Animal Welfare and Ethical Review Body of the Wellcome Trust Genome Campus.

### Isolation of mTEC cells

Thymi were collected from 2- and 4-week-old wild-type C57BL/6 male and female mice. Epithelial cell isolation was performed based on^64^. Up to 3 thymi were cleaned of fat and connective tissue, finely minced and pooled together. Thymocytes were flushed by gentle agitation with a magnetic stirrer in RPMI-1640 for 30 min, at 4 °C. Thymic fragments were recovered by settling, and the supernatant discarded. After further dispersion, three additional washes were performed. Fragments were then incubated in 5 mL of 0.125% (w/v) Collagenase D and 0.1% (w/v) DNAse I (both from Roche) in RPMI-1640, at 37 °C for 15 min, with gentle pipetting every 5 min. The supernatant was collected and kept on ice, while the thymic fragments were subject to two further incubations. The remaining fragments were finally resuspended in 5 ml of 0.125%(w/v) Collagenase/Dispase (Roche) and 0.1%(w/v) DNaseI in RPMI-1640 for 30 min at 37 °C, with gentle agitation every 15 min. All the collected fractions were pooled, centrifuged at 450 × g for 5 min and incubated in 5 mM EDTA, 1% FCS, 0.02% (w/v) NaN_3_ in PBS (EDTA/FACS buffer) for 10 min at 4 °C. After filtering through a 100μm-strainer, the resulting cell suspension was depleted of hematopoietic cells by Magnetic-Activated Cell Sorting (MACS) using CD45-MicroBeads (Miltenyi Biotec). For sorting, the recovered fraction was blocked using anti-CD16/CD32 (clone 2.4G2, Tonbo) and then stained using anti-CD45-PerCP-Cy5.5 (clone 30-F11, BioLegend), anti-Ly-51-FITC (clone 6C3, BioLegend), anti-CD326(Ep-CAM)-AF647 (clone G8.8, BioLegend), UEA-1-Biotin and Streptavidin-Pacific Blue. mTECs (CD45^−^Ly-51^−^UEA^+^) were sorted with a MoFlo™ XDP (Beckman Coulter, Inc.). Propidium iodide was used as a viability dye.

### Single-cell mRNA sequencing

Single cell capture and processing for the main dataset was performed using the Fluidigm C1 system as in ref.^65^. The mTEC suspension obtained from sorting was loaded onto the Fluidigm C1 platform using medium–sized capture chips (10–17μm cells). One μl of a 1:400 or 1:2000 dilution of External RNA Control Consortium (ERCC) spike-ins (Ambion, Life Technologies) were included in the lysis buffer. Reverse transcription and cDNA preamplification were performed using the SMARTer Ultra Low RNA kit (Clontech). In total, three C1 runs were performed: one with cells from 2-week old mice and, on a separate day, two parallel runs with cells from 4-week old mice. The cDNA libraries for sequencing were prepared using Nextera XT DNA Sample Preparation Kit (Illumina), according to the protocol supplied by Fluidigm (PN 100–5950 B1). Libraries from 96 single cells were pooled and subsequently purified using AMPure XP beads (Beckman Coulter). Pooled samples were sequenced on an Illumina HiSeq. 2500 instrument, using paired-end 100-base pair reads.

For the plate-based Smart-seq. 2 dataset, single cells were sorted in 2uL of Lysis Buffer (1:20 solution of RNase Inhibitor (Clontech or Invitrogen) in 0.2% v/v Triton X-100 (Sigma-Aldrich) in 96 well plates, spun down and immediately frozen at −80 degrees. Oligo-dT primer, dNTPs (ThermoFisher) and ERCC RNA Spike-In Mix (1:50,000,000 final dilution, Ambion) were then added, and Reverse Transcription and PCR were performed as in ref.^33^.

DNA was subjected to quality control using 1 µl of amplified DNA on an Agilent 2100 BioAnalyzer (Agilent Technologies, Santa Clara, CA, USA) using the Agilent High Sensitivity DNA kit. Of plates that pass quality control, 5 µl of DNA were cleaned using Agencourt AMPure XP beads (Beckman Coulter) at a 1.0× ratio on a Hamilton STAR (Hamilton Robotics) liquid handler, eluted in 25 µl buffer EB (Qiagen) and transferred to LabCyte 384_PP acoustic plates (LabCyte). DNA was quantified using the AccuClear Ultra High Sensitivity dsDNA quantification kit (Biotium). Samples were normalised to a concentration of 1 ng/µl before library preparation using a modified Illumina Nextera DNA library preparation kit (Illumina). In brief, 500 nl of normalised cDNA samples were tagmented by adding 100 nl Tn5-buffer mix and incubating for 5 min at 55 °C. Tagmentation reactions were neutralised by adding a total concentration of 0.2% sodium dodecyl sulfate (Sigma-Aldrich). 125 nl of in-house index adapters (Integrated DNA Technologies) similar to Illumina N7 and N5 indices were added to the tagmentation reaction before adding 1.5 µl of KAPA HiFi DNA polymerase (KAPA Biosystems) and performing 12 cycles of PCR according to the manufacturer’s instructions. After PCR, all samples were pooled into 288-plex pools using a Hamilton STAR liquid handler and the pool cleaned using Agencourt AMPure XP beads at a 0.6× ratio. Library pools were eluted in buffer EB and quality controlled using an Agilent 2100 BioAnalyzer and Agilent High Sensitivity DNA kits before adjusting the concentration to 10 nM and performing a 1:1000 dilution using a Hamilton STAR liquid handler. The diluted pools were quantified using the KAPA qPCR library quantification kit on a Roche LightCycler 480 (Roche) before a final dilution to 4 nM.

### Processing and quality control of single-cell mRNA-seq data

Reads were mapped to the *Mus musculus* genome (Ensembl version 38.75) concatenated with the ERCC sequences, using GSNAP (version 2014-05-15_v2, ref.^66^) with default parameters. The read counts for each gene were determined using HTseq (version 2.6.0, ref.^67^), and TPM calculated. Only genes expressed with 5 or more TPM in at least 5 cells across all datasets were kept. As cell quality control measures, cells with fewer than 1000 genes, fewer than 500,000 reads mapping to exons or with more than 20% reads mapped to mitochondrial genes were excluded from further analyses.

Cyclone package^28^ was used to determine the cell cycle phase of each cell. scLVM package^27^ was run for all datasets, and the corrected matrices were used for the datasets showing relevant number of cycling cells, *i*.*e*. Sansom *et al*. and Brennecke *et al*. datasets^16,25^. For our own dataset, which presented a very limited number of cycling cells, we chose not to use scLVM as it introduced additional confounding factors to our analysis: the effect of cell size (number of genes detected) was spread across multiple PC1s (Supplementary Figure 5A), while it exhibited strong negative correlation with PC1 in the original matrix (Spearman correlation −0.92). Nevertheless, cell consensus clustering (see below) was also performed with the corrected matrix to confirm that our three clusters were not affected by cell cycle.

### Differential expression

For DE analysis, two linear models were fitted to the expression levels of each gene separately: a full model containing the information for each mTEC subpopulation and a reduced model only including an intercept term. These were then compared by a likelihood-ratio test, and *p*-values were adjusted to account for the false discovery rate associated to multiple testing.

### Consensus Clustering

For cell clustering, the genes contributing the most to PC2 and PC3 in our main dataset PCA were used (|gene loading| > 0.02, Supplementary Table 4). For each dataset, clusters were determined using the ConsensusClusterPlus package^68^ with 70% cell and gene resampling, in 2000 resampling events. Although up to 6 clusters were explored per dataset, 3 clusters represented the most stable option in all cases.

### Genomic clustering

A nearest-neighbour method was used to assess genomic clustering of expressed TRAs. First, we determined a set of genes with a similar distance distribution for each TRA subset, minimizing the Kullback-Leibler (KL) divergence between them in an iterative manner. The residual divergence between these distributions (KLgen) will later be taken into account. Then, for each cell, we calculated the distance between each expressed TRA gene and its closest expressed TRA. In parallel, we sampled a similar number of expressed control genes and measured their distances to the nearest expressed neighbour control gene. This sampling step was repeated a thousand times per cell and these distances were used as background. To compare the mean distance in both distributions we used the Mann-Whitney-Wilcoxon test, and to quantify how similar the distributions were, we calculated their KL divergence and subtracted KLgen. This analysis was conducted per mTEC sub-population and for each TRA subset, *i*.*e*. *Aire*-dependent, *Aire*-enhanced and *Aire*-unnaffected TRAs.

### Pseudotime inference

Pseudotime inference was performed independently in three different settings: 1. PCA was used as dimension reduction method, and R package pseudogp^34^ (Campbell and Yau 2016) was used to infer trajectory based on the PCA results; 2. R package Destiny^35^ was used to reduce dimension of the data with diffusion maps, and pseudogp was used to infer trajectory; 3. R package Monocle^36,69^ was used to infer trajectory with the original high dimensional data as input. We used the Monocle2^36^ release, performing the analysis using the normalized data (TPM) and all expressed genes. The direction of pseudotime was inferred from expression patterns of known markers of jTECs and mTECs.

### Binding motif enrichment analysis

We performed binding motif enrichment analysis on the genes which are correlated with PC2 or PC3 in Fig. 2A (Supplementary Table 4). Genes which have loadings greater than 0.02 on PC2 or PC3 were selected and input to the gprofile function in the R package gProfileR^70^, using its default settings. The function gprofile outputs the enriched TF families and corresponding target genes from our input gene set. For each pair of TF and target gene, we calculate Spearman correlation and Jaccard Index. The Jaccard Index was calculated based on binarized gene expression levels. Network visualisation was created using TF-target pairs with Spearman correlation |r| > 0.3 (p-value < 0.005) and Jaccard Index j > 0.3 (Fig. 3C).

## Electronic supplementary material

Supplementary Information

Supplementary table 1

Supplementary table 2

Supplementary table 3

Supplementary table 4
