# Supplementary material for: Single-cell RNA-sequencing resolves self-antigen expression during mTEC development
Source: Sci Rep. 2018 Jan 12;8:685. doi: 10.1038/s41598-017-19100-4 (PMC5766627; doi:10.1038/s41598-017-19100-4)
Supplement: Supplementary file 1 — Supplementary Information [file 41598_2017_19100_MOESM1_ESM.pdf]

Supplementary information

Single-cell RNA-sequencing resolves self-antigen expression during mTEC development

Ricardo J Miragaia[1,2,3,#], Xiuwei Zhang[1,4,#], Tomás Gomes[2], Valentine Svensson[1], Tomislav Ilicic[2], Johan Henriksson[1], Gozde Kar[1], Tapio Lönnberg[1,2,5]*

[1]European Bioinformatics Institute (EMBL-EBI), European Molecular Biology Laboratory, Wellcome Trust Genome Campus, Hinxton, Cambridge CB10 1SD, United Kingdom

[2]Wellcome Trust Sanger Institute, Wellcome Trust Genome Campus, Hinxton, Cambridge CB10 1SA, United Kingdom

[3]Centre of Biological Engineering, University of Minho, Campus de Gualtar, 4710-057 Braga, Portugal

[4]Current affiliation: University of California, Berkeley, USA

[5]Current affiliation: Turku Centre for Biotechnology, University of Turku and Åbo Akademi University, Turku, Finland

[#]With equal contribution

[*]Corresponding author

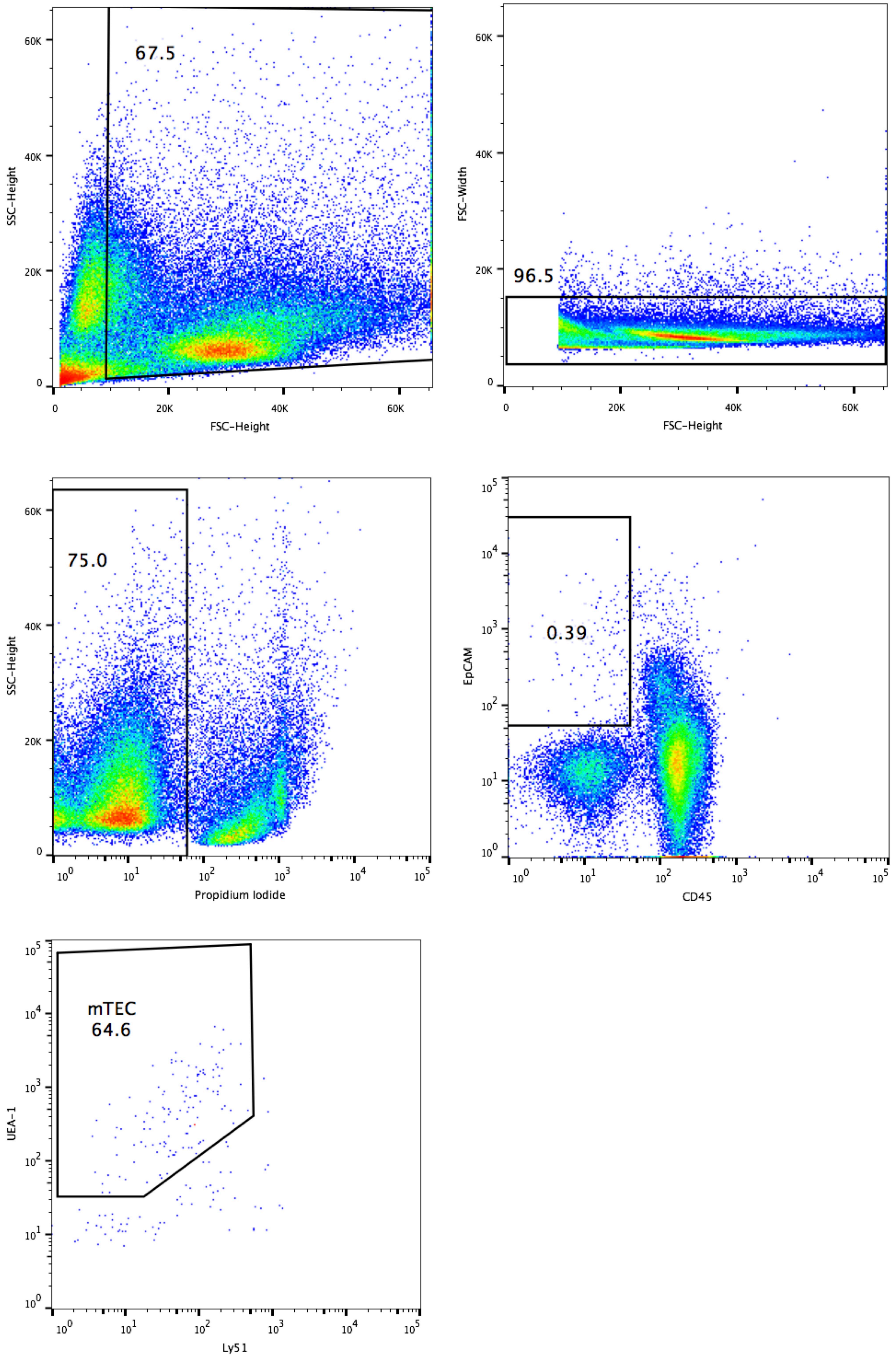
67.5
SSC-Height
FSC-Height
96.5
FSC-Width
FSC-Height
75.0
SSC-Height
Propidium Iodide
0.39
EpCAM
CD45
mTEC
64.6
UEA-1
Ly51

Supplementary figure 1. Flow cytometry cell sorting strategy for isolation of mTECs.

mTECs were sorted as $PI^{-}CD45^{-}EpCAM^{+}Ly51^{-}UEA\text{-}1^{+}$ using a MoFlo™ XDP (Beckman Coulter, Inc.).

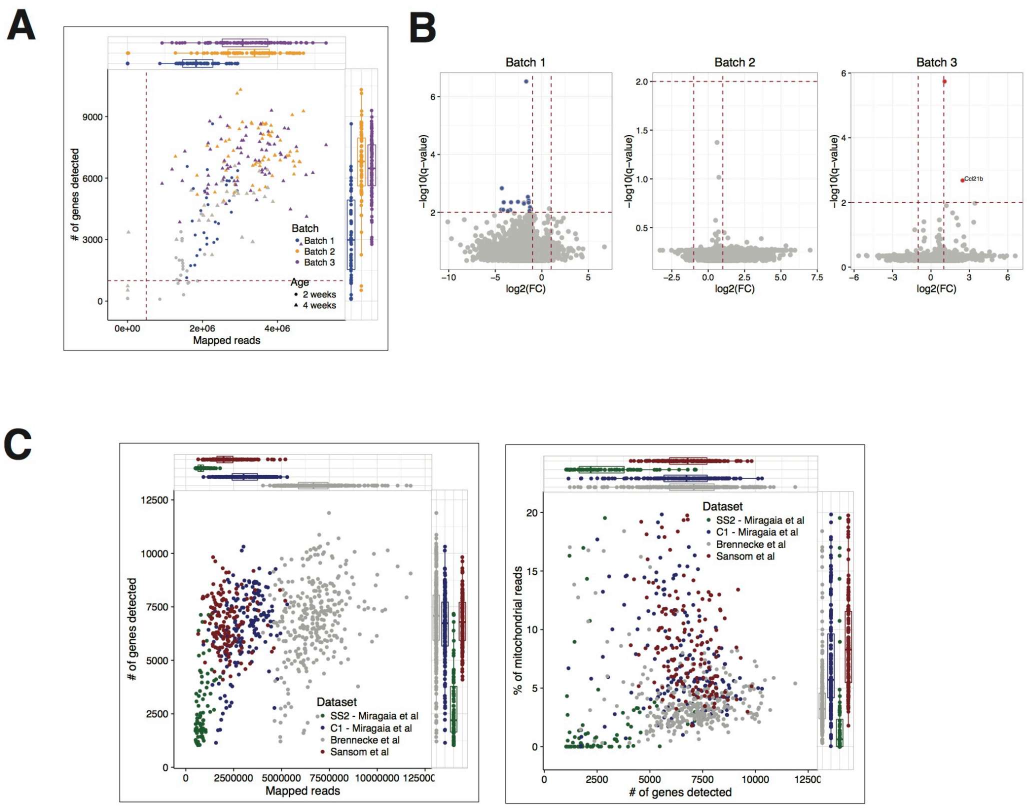

Supplementary figure 2. scRNA-seq quality control.

A) Number of mapped reads and genes detected in our C1 dataset. Cells with less than 1000 genes, less than 500,000 mapped reads or more than 20% of mitochondrial reads were excluded (gray symbols).

B) Differential expression between different batches within our C1 dataset.

C) QC metrics for all datasets used. Same thresholds were used across all datasets.

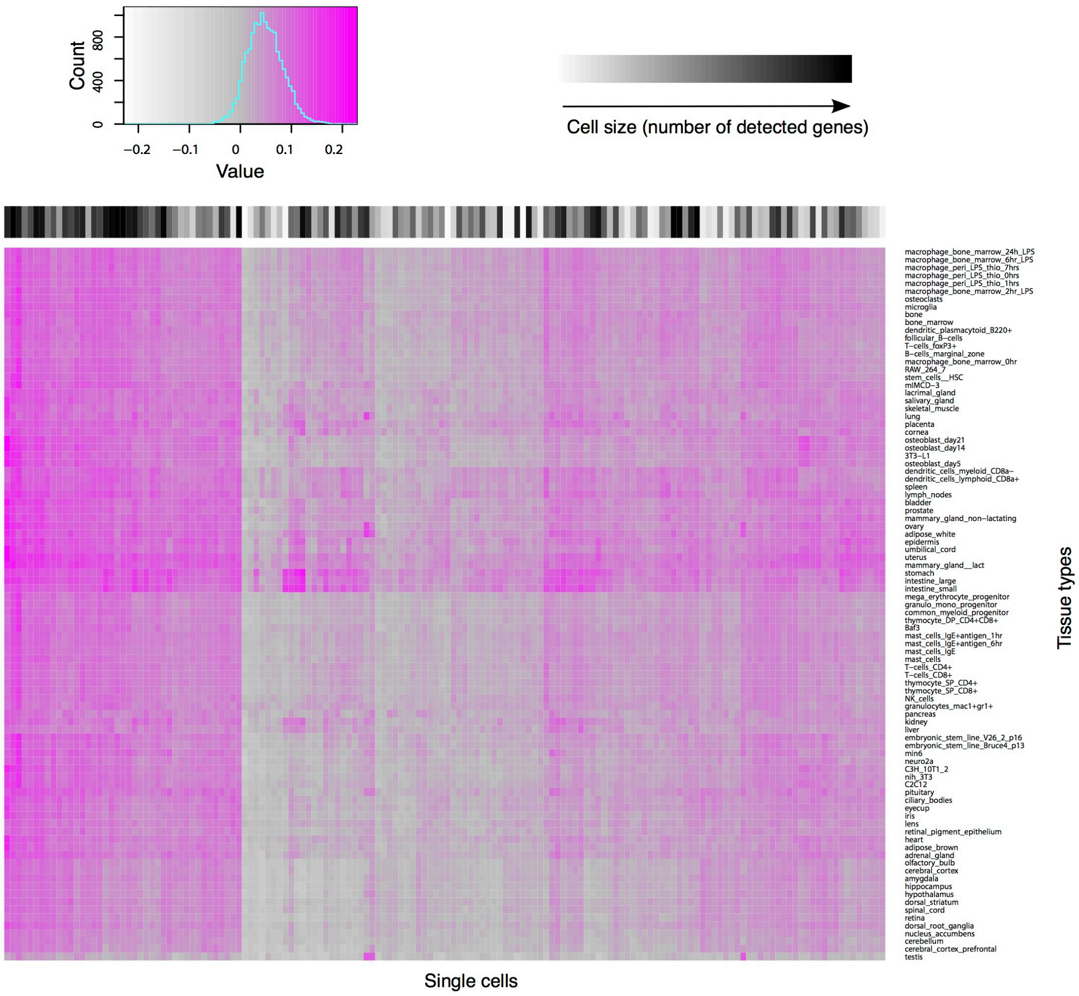

Supplementary figure 3. Correlation between individual mTECs and tissues.

Correlations between individual mTECs and microarray data from multiple tissues [71] were measured and clustered. All expressed genes were used in the calculations. Colour bar denotes cell size, as estimated by number of detected genes.

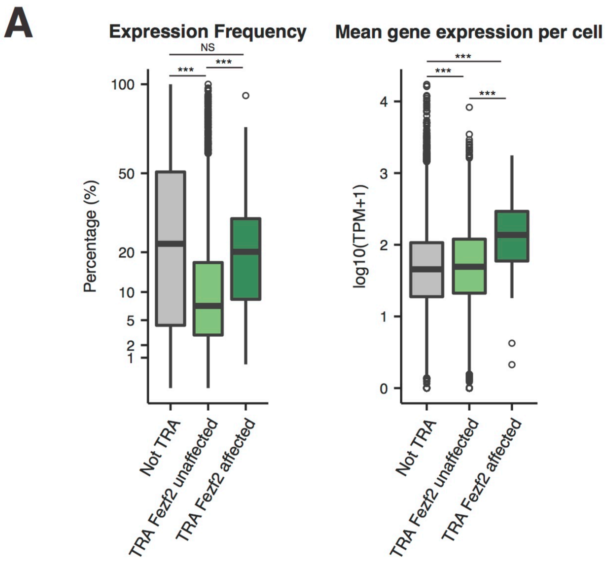

Supplementary figure 4. Expression of Fezf2-regulated TRAs at the single-cell level.

Comparing TRAs from different Fezf2 categories in terms of expression frequency and mean expression level across all cells. *** p-value<0.001, ** p-value<0.01, * p-value<0.05, NS – not significant, according to Mann-Whitney-Wilcoxon test, p-value adjusted using Bonferroni correction.

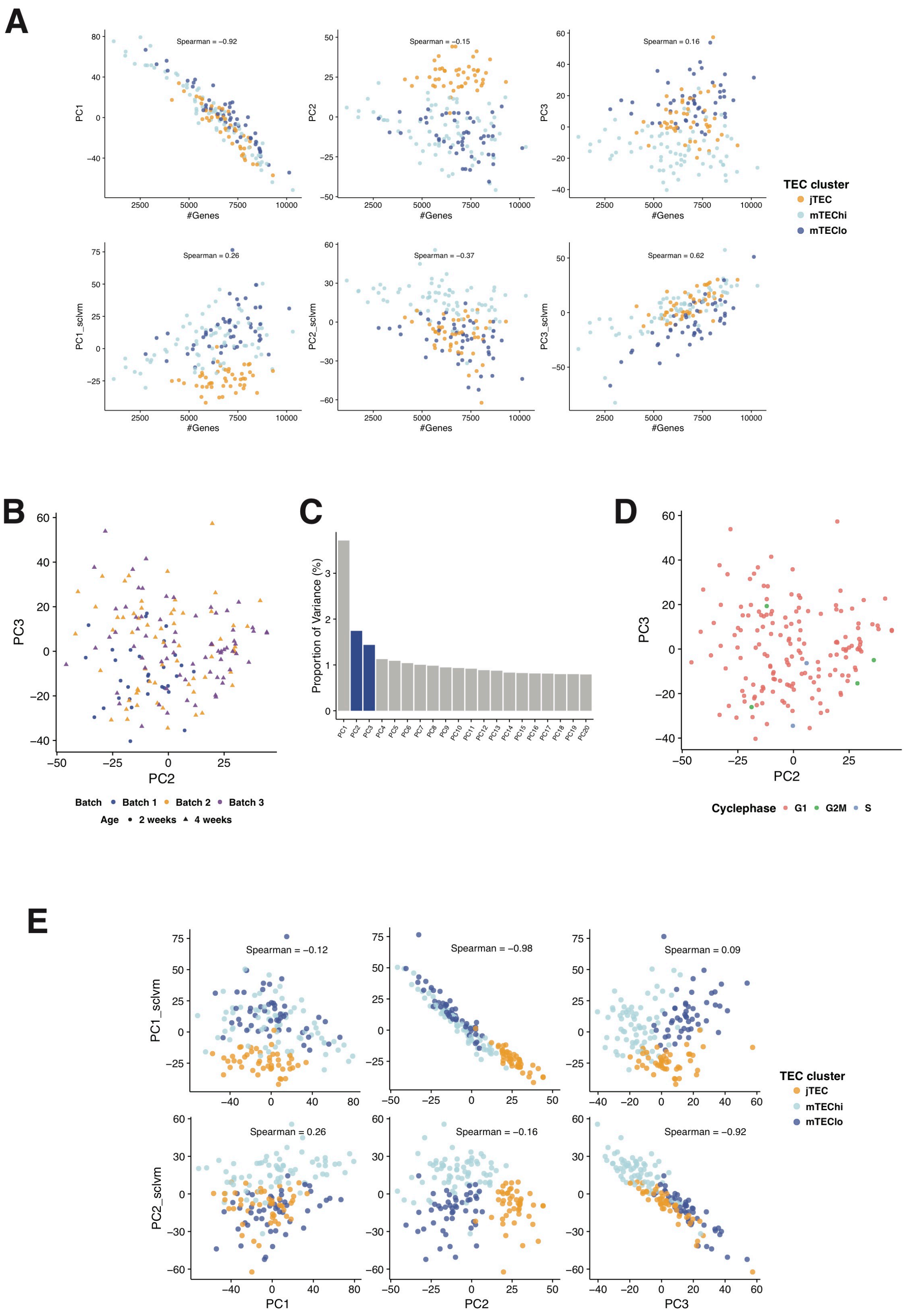

A
Spearman = −0.92
Spearman = −0.15
Spearman = 0.16
Spearman = 0.26
Spearman = −0.37
Spearman = 0.62
PC1
PC2
PC3
PC1_sclvm
PC2_sclvm
PC3_sclvm
#Genes
TEC cluster
jTEC
mTEChi
mTEClo
B
PC3
PC2
Batch
Batch 1
Batch 2
Batch 3
Age
2 weeks
4 weeks
C
Proportion of Variance (%)
PC1
PC2
PC3
PC4
PC5
PC6
PC7
PC8
PC9
PC10
PC11
PC12
PC13
PC14
PC15
PC16
PC17
PC18
PC19
PC20
D
PC3
PC2
Cyclephase
G1
G2M
S
E
Spearman = −0.12
Spearman = −0.98
Spearman = 0.09
Spearman = 0.26
Spearman = −0.16
Spearman = −0.92
PC1_sclvm
PC2_sclvm
PC1
PC2
PC3
TEC cluster
jTEC
mTEChi
mTEClo

Supplementary figure 5. Principal Component Analysis of in-house C1 dataset.

A) Correlation between the first three PCs and the number of detected genes per cell, either in uncorrected (top) or scLVM corrected data (bottom).
B) Distribution of the three C1 batches along Principal Component 2 (PC2) and PC3.
C) Proportion of variance (%) for the first 20 PCs. PC2 and PC3 seem to contain most biological variance.
D) Cell cycle classification of single-cells (G1/G0, G2M, S) as determined by the Cyclone package.
E) Correlation between PC1 and PC2 in scLVM corrected data and PC1, PC2, PC3 in the uncorrected data.

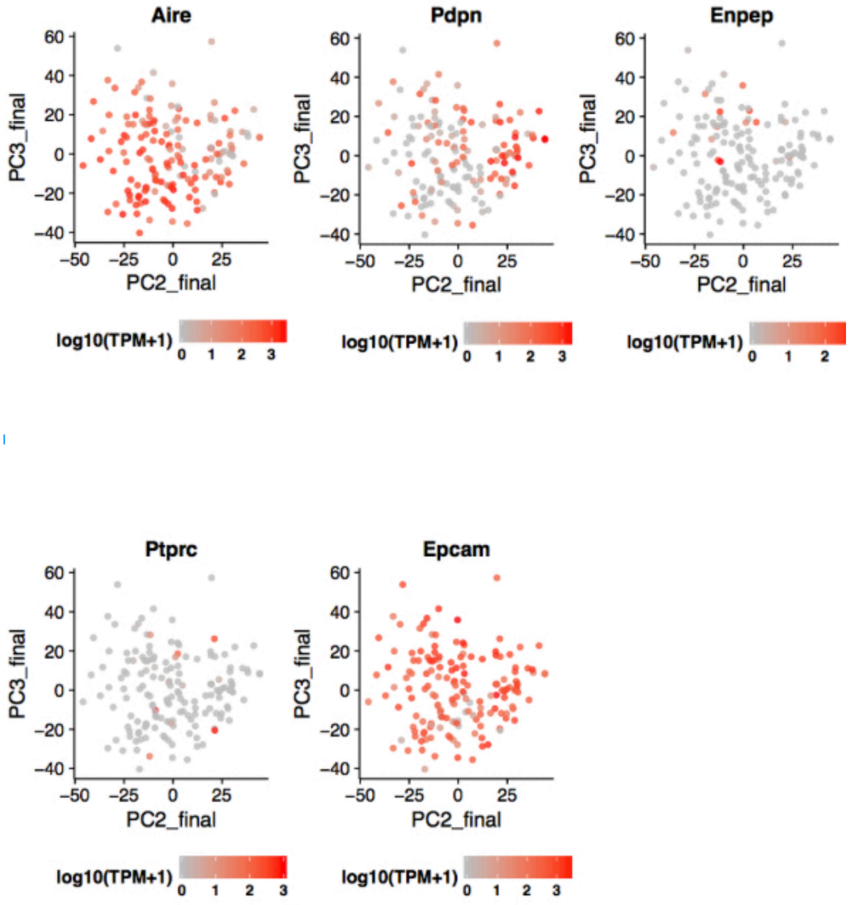

Supplementary figure 6. Expression of key cell type-associated marker genes overlaid on the Principal Component Analysis.

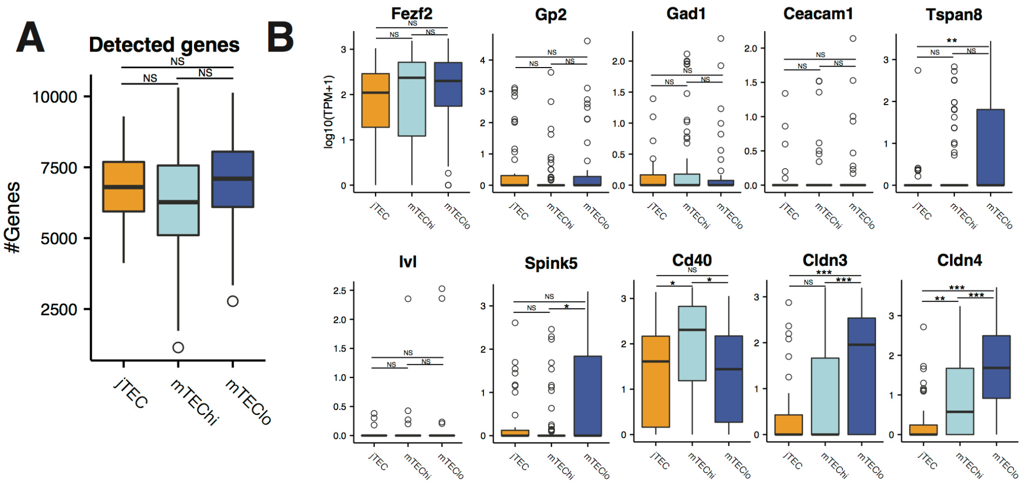

Supplementary figure 7. Subpopulation comparisons for in-house C1 dataset.

A) Number of genes detected per subpopulation C1 dataset.

B) Expression of selected marker genes in the jTEC, mTEChi, and mTEClo populations.

*** p-value<0.001, ** p-value<0.01, * p-value<0.05, NS – not significant, according to Mann-Whitney-Wilcoxon test, p-value adjusted using Bonferroni correction.

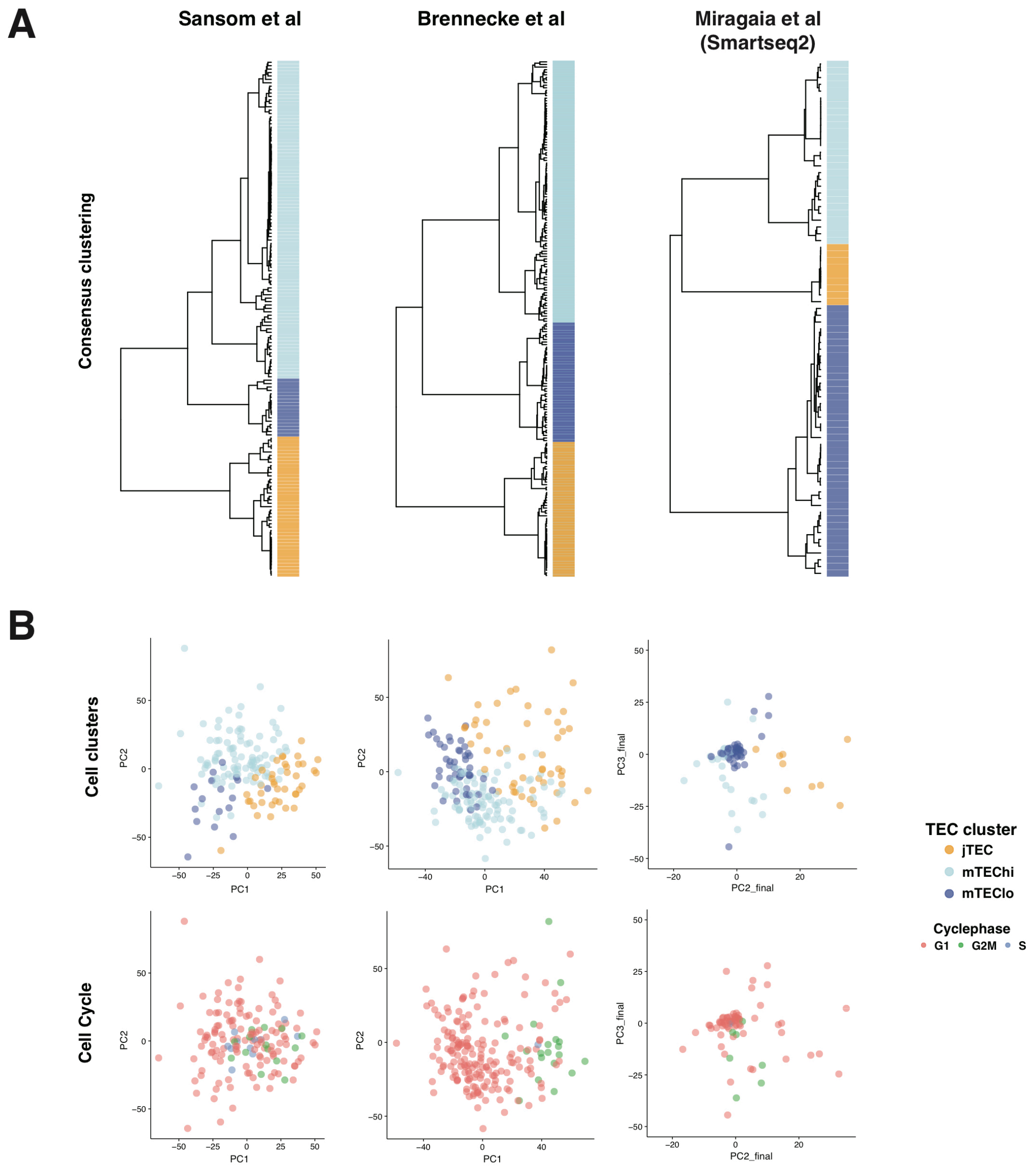

Supplementary figure 8. PCA on publicly available and in-house Smartseq2 mTEC datasets.

A) Clustering dendrograms for the Sansom et al, Brennecke et al and in-house Smartseq2 datasets.

B) PCA of Sansom et al, Brennecke et al and in-house Smartseq2 datasets, coloured by cell clusters as determined by hierarchical clustering (top). Sansom et al and Brennecke et al datasets were subject to scLVM correction (see text). From the Miragaia et al. PCA visualization, two outlier cells were removed in the interest of clarity. PCA coloured by cell cycle, as determined by the Cyclone package (bottom).

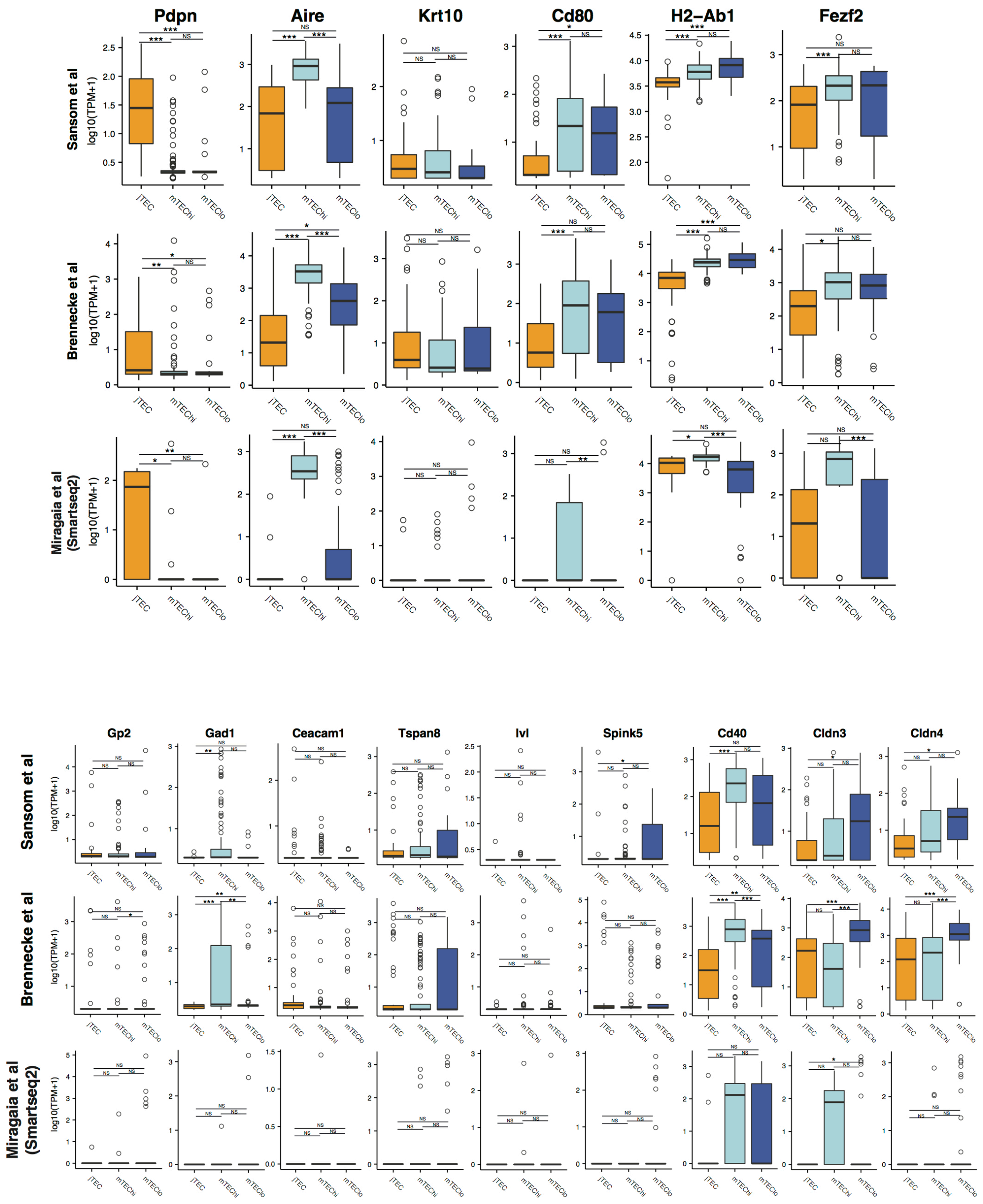

Supplementary figure 9. Differential expression of selected marker genes across mTEC subpopulations in publicly available and in-house Smartseq2 mTEC datasets.

*** p-value<0.001, ** p-value<0.01, * p-value<0.05, NS – not significant, according to Mann-Whitney-Wilcoxon test, p-value adjusted using Bonferroni correction.

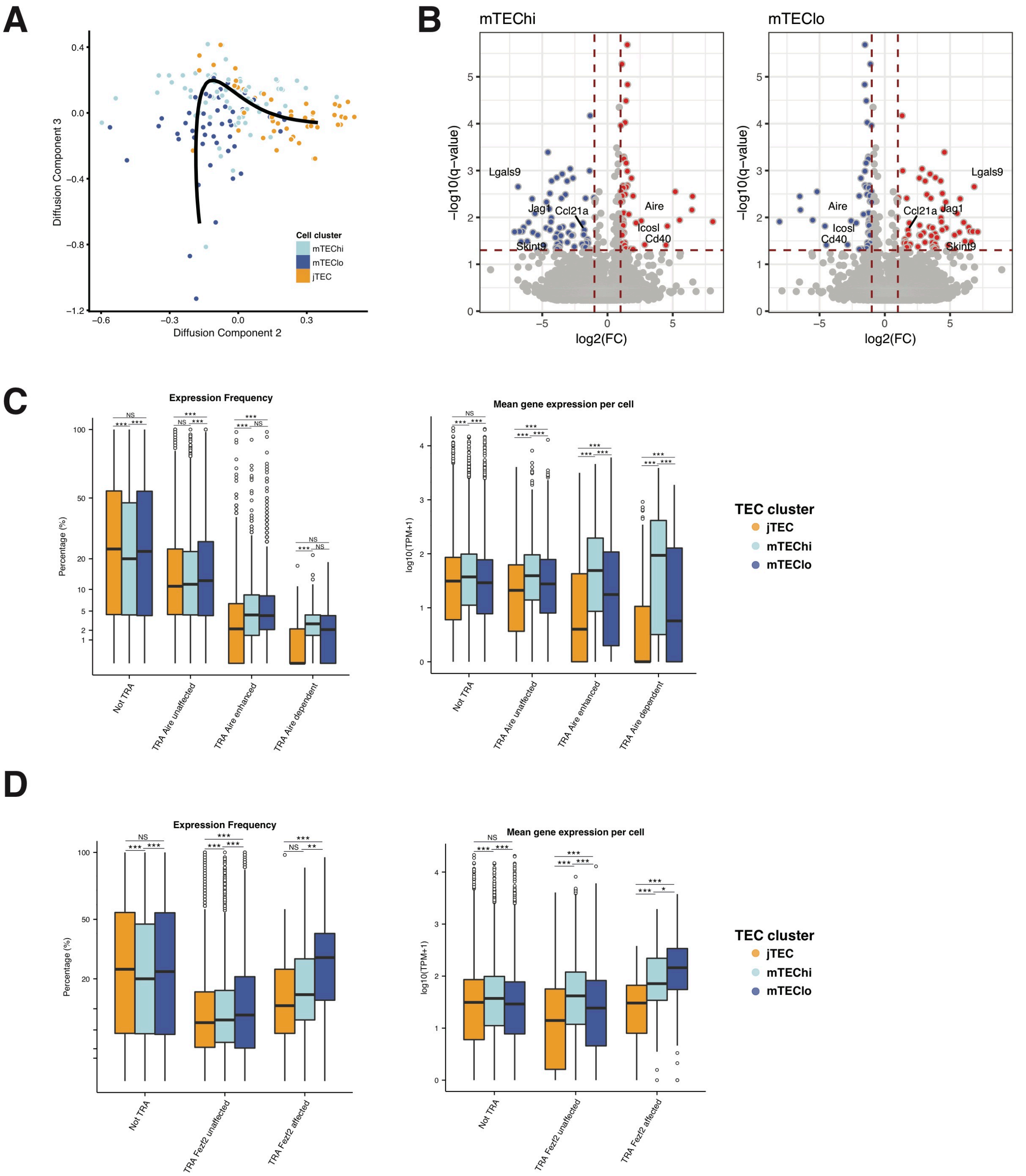

Supplementary figure 10. Comparisons of mTEC subpopulations.

A) Pseudotemporal ordering of single cells using dimensionality reduction based on diffusion maps[35] and trajectory inference performed with Pseudogp [34].

B) Genes differentially expressed (DE) between mTEChi and mTEC lo subpopulations. The significance of this DE was calculated using a linear model. q-value < 0.01 and |FC| > 1.

C) TRA expression sectioned by Aire and Fezf2 dependency (D) and mTEC subpopulations in C1 dataset. For each category, gene expression frequency and mean expression level were

calculated across all cells. *** p-value<0.001, ** p-value<0.01, * p-value<0.05, NS – not significant, according to Mann-Whitney-Wilcoxon test, p-value adjusted using Bonferroni correction.

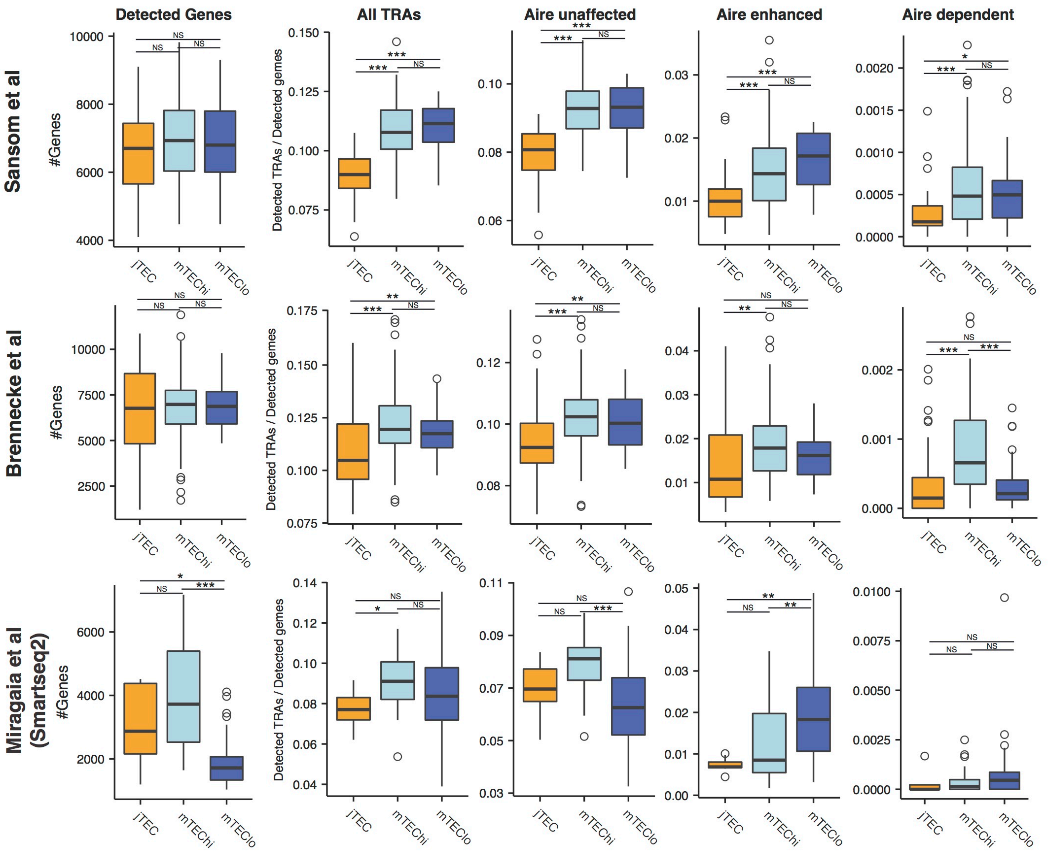

Supplementary figure 11. Publicly available and in-house Smartseq2 mTEC datasets.

A) Number of genes detected per mTEC subpopulation in each dataset.
B) Number of TRA genes expressed in the jTEC, mTEChi, and mTEClo populations, in each dataset. To account for differences in library sizes, the number of detected TRA genes were normalised to the number of detected genes per cell.

*** p-value<0.001, ** p-value<0.01, * p-value<0.05, NS – not significant, according to Mann-Whitney-Wilcoxon test, p-value adjusted using Bonferroni correction.

Supplementary tables (external files)

Supplementary table 1. Single-cell gene expression data normalized as transcripts per million (TPM).

Supplementary table 2. Annotation information for single cells.

Supplementary table 3. Genes specifically expressed in jTEC, mTEChi and mTEClo subpopulations.

Supplementary table 4. Genes contributing the most to PC2 and PC3 in our main dataset PCA (|gene loading| > 0.02) and used for consensus clustering of the single cells.
